# Risk for Newly Diagnosed Diabetes >30 Days After SARS-CoV-2 Infection Among Persons Aged <18 Years — United States, March 1, 2020–June 28, 2021

**DOI:** 10.15585/mmwr.mm7102e2

**Published:** 2022-01-14

**Authors:** Catherine E. Barrett, Alain K. Koyama, Pablo Alvarez, Wilson Chow, Elizabeth A. Lundeen, Cria G. Perrine, Meda E. Pavkov, Deborah B. Rolka, Jennifer L. Wiltz, Lara Bull-Otterson, Simone Gray, Tegan K. Boehmer, Adi V. Gundlapalli, David A. Siegel, Lyudmyla Kompaniyets, Alyson B. Goodman, Barbara E. Mahon, Robert V. Tauxe, Karen Remley, Sharon Saydah

**Affiliations:** ^1^CDC COVID-19 Emergency Response Team; ^2^Division of Diabetes Translation, National Center for Chronic Disease Prevention and Health Promotion, CDC.

The COVID-19 pandemic has disproportionately affected people with diabetes, who are at increased risk of severe COVID-19.* Increases in the number of type 1 diabetes diagnoses (*1*,*2*) and increased frequency and severity of diabetic ketoacidosis (DKA) at the time of diabetes diagnosis (*3*) have been reported in European pediatric populations during the COVID-19 pandemic. In adults, diabetes might be a long-term consequence of SARS-CoV-2 infection (*4*–*7*). To evaluate the risk for any new diabetes diagnosis (type 1, type 2, or other diabetes) >30 days^†^ after acute infection with SARS-CoV-2 (the virus that causes COVID-19), CDC estimated diabetes incidence among patients aged <18 years (patients) with diagnosed COVID-19 from retrospective cohorts constructed using IQVIA health care claims data from March 1, 2020, through February 26, 2021, and compared it with incidence among patients matched by age and sex 1) who did not receive a COVID-19 diagnosis during the pandemic, or 2) who received a prepandemic non–COVID-19 acute respiratory infection (ARI) diagnosis. Analyses were replicated using a second data source (HealthVerity; March 1, 2020–June 28, 2021) that included patients who had any health care encounter possibly related to COVID-19. Among these patients, diabetes incidence was significantly higher among those with COVID-19 than among those 1) without COVID-19 in both databases (IQVIA: hazard ratio [HR] = 2.66, 95% CI = 1.98–3.56; HealthVerity: HR = 1.31, 95% CI = 1.20–1.44) and 2) with non–COVID-19 ARI in the prepandemic period (IQVIA, HR = 2.16, 95% CI = 1.64–2.86). The observed increased risk for diabetes among persons aged <18 years who had COVID-19 highlights the importance of COVID-19 prevention strategies, including vaccination, for all eligible persons in this age group,^§^ in addition to chronic disease prevention and management. The mechanism of how SARS-CoV-2 might lead to incident diabetes is likely complex and could differ by type 1 and type 2 diabetes. Monitoring for long-term consequences, including signs of new diabetes, following SARS-CoV-2 infection is important in this age group

Retrospective cohorts were constructed using two U.S. medical claims databases: IQVIA^¶^ and HealthVerity.** Patients who were aged <18 years on their index encounter date and who were continuously enrolled in a closed payor system throughout the study period^††^ were followed from their index date^§§^ until the end of the study period. Patients were excluded from the analysis if they had preexisting diabetes, defined as one or more *International Classification of Diseases, Tenth Revision, Clinical Modification* (ICD-10-CM) diagnosis codes for diabetes (E08–E13) in the 1–13 months preceding their index date.

In the IQVIA database, patients with a COVID-19 diagnosis (ICD-10-CM codes B97.29 or U07.1)^¶¶^ during March 1, 2020–February 26, 2021, were defined as having COVID-19. Patients with COVID-19 were matched by age and sex to pandemic and prepandemic period comparison groups.***The pandemic period non–COVID-19 group comprised patients without COVID-19–related ICD-10-CM codes during March 1, 2020–February 26, 2021.^†††^ The prepandemic period ARI group comprised patients with a diagnosis of ARI^§§§^ (Supplementary Table 1, https://stacks.cdc.gov/view/cdc/113062) during March 1, 2017–February 26, 2018. A prepandemic non-ARI group consisted of those in this age group whose records did not include ARI ICD-10-CM codes during March 1, 2017–February 26, 2018.

In HealthVerity, the COVID-19 group comprised patients aged <18 years whose record included an ICD-10-CM diagnosis code for COVID-19 or a positive SARS-CoV-2 polymerase chain reaction (PCR) test result during March 1, 2020–June 28, 2021. The pandemic period non–COVID-19 group consisted of those who had a negative SARS-CoV-2 PCR test result and no record of COVID-19 diagnosis codes or positive SARS-CoV-2 test results during the same period. Both groups were identified within a subset of CDC-licensed HealthVerity data that includes patients with a health care encounter possibly related to COVID-19 (Supplementary Table 2, https://stacks.cdc.gov/view/cdc/113062). There was no prepandemic comparison period for the HealthVerity data.

Incident diabetes was defined as one or more health care claims with a diabetes diagnosis (ICD-10-CM codes E08–E13) occurring >30 days after the index date (excluding cases of transient, resolved hyperglycemia). Frequencies of incident diabetes codes on, and DKA codes on or before, the date of the incident diabetes encounter were calculated.^¶¶¶^ Cox regression models were used to estimate HRs for diabetes risk. HRs were also estimated by age group and sex. Age and sex effect modifications were assessed using interaction terms. SAS (version 9.4; SAS Institute) and PANDAS (version 1.3.0; PANDAS Community) software were used to conduct all analyses. This activity was reviewed by CDC and was conducted consistent with applicable federal law and CDC policy.****

Among 80,893 patients with COVID-19 in the IQVIA database, the mean age was 12.3 years, 50.1% were female, and 0.7% were hospitalized at their index COVID-19 encounter (Table 1). Among 439,439 patients with COVID-19 in HealthVerity, the mean age was 12.7 years, 50.1% were female, and 0.9% were hospitalized at their index encounter. Diabetes was coded in 0.08% (IQVIA) and 0.25% (HealthVerity) of claims for patients with COVID-19, with the majority of diabetes diagnoses for type 1 or type 2 (IQVIA, 94.1%; HealthVerity, 94.0%). In comparison, 0.03% (IQVIA) and 0.19% (HealthVerity) diabetes cases were coded among those without COVID-19. DKA was reported in 48.5% (IQVIA) and 40.2% (HealthVerity) of patients with COVID-19 and diabetes; these proportions were higher than DKA reported in patients with diabetes without COVID (IQVIA: non-COVID 13.6%; ARI 22.0%; non-ARI 27.5%; HealthVerity: 29.7%).

**TABLE 1:** Characteristics of matched pediatric groups with and without evidence of COVID-19 or acute respiratory infection and number of new diabetes diagnoses, by age, sex, and preceding COVID-19 or acute respiratory infection diagnosis — IQVIA PharMetrics Plus and HealthVerity claims databases, United States, March 1, 2020–June 28, 2021*

Database/Characteristic	No. (%)				
	Pediatric overall	COVID-19	Non–COVID-19	ARI	Non-ARI
**IQVIA**					
**Total no. of patients**	1,698,753	80,893	404,465	404,465	808,930
**Age, mean (SD), yrs**	12.3 (4.3)	12.3 (4.3)	12.3 (4.3)	12.3 (4.3)	12.3 (4.3)
**Age group, yrs**					
0–4	124,530 (7.3)	5,930 (7.3)	29,650 (7.3)	29,650 (7.3)	59,300 (7.3)
5–11	483,273 (28.4)	23,013 (28.4)	115,065 (28.4)	115,065 (28.4)	230,130 (28.4)
12–15	592,830 (34.9)	28,230 (34.9)	141,150 (34.9)	141,150 (34.9)	282,300 (34.9)
16–17	498,120 (29.3)	23,720 (29.3)	118,600 (29.3)	118,600 (29.3)	237,200 (29.3)
**Female sex**	850,857 (50.1)	40,517 (50.1)	202,585 (50.1)	202,585 (50.1)	405,170 (50.1)
**Hospitalized at index encounter**	6,473 (0.4)	566 (0.7)	614 (0.2)	1,602 (0.4)	3,691 (0.5)
**New diabetes diagnosis^†^**					
**Overall**	**937 (0.06)**	**68 (0.08)**	**132 (0.03)**	**227 (0.06)**	**510 (0.06)**
DM type (% of all newly diagnosed diabetes)^§^					
Type 1 or Type 2	891 (95.1)	64 (94.1)	124 (93.9)	210 (92.5)	493 (96.7)
Due to underlying condition/Other	31 (3.3)	3 (4.4)	6 (4.5)	8 (3.5)	14 (2.7)
Drug or chemical induced	15 (1.6)	1 (1.5)	2 (1.5)	9 (4.0)	3 (0.6)
DKA (% of all newly diagnosed diabetes)^¶^	241 (25.7)	33 (48.5)	18 (13.6)	50 (22.0)	140 (27.5)
**HealthVerity**					
**Total no. of patients**	878,878	439,439	439,439	—**	—
**Age, mean (SD), yrs**	12.7 (3.8)	12.7 (3.8)	12.7 (3.8)	—	—
Age group, yrs					
0–4	28,532 (3.2)	14,266 (3.2)	14,266 (3.2)	—	—
5–11	321,496 (36.6)	160,748 (36.6)	160,748 (36.6)	—	—
12–15	319,458 (36.3)	159,729 (36.3)	159,729 (36.3)	—	—
16–17	209,392 (23.8)	104,696 (23.8)	104,696 (23.8)	—	—
**Female sex**	440,024 (50.1)	220,012 (50.1)	220,012 (50.1)	—	—
**Hospitalized at index encounter**	13,118 (3.0)	7,510 (0.9)	5,608 (1.3)	—	—
**New diabetes diagnosis**					
**Overall**	**1,973 (0.22)**	**1,120 (0.25)**	**853 (0.19)**	**—**	**—**
DM type (% of all newly diagnosed diabetes)					
Type 1 or type 2	1,871 (94.8)	1,053 (94.0)	818 (95.9)	—	—
Due to underlying condition/Other	67 (3.4)	42 (3.8)	25 (2.9)	—	—
Drug or chemical induced	35 (1.8)	25 (2.2)	10 (1.2)	—	—
DKA (% of all newly diagnosed diabetes)	703 (35.6)	450 (40.2)	253 (29.7)	—	—

**Abbreviations:** ARI = acute respiratory infection; DKA = diabetic ketoacidosis; DM = diabetes mellitus; ICD-10-CM = *International Classification of Diseases, Tenth Revision, Clinical Modification*.

* Groups in IQVIA included patients aged <18 years with or without COVID-19 (COVID-19; non–COVID-19, respectively) and patients aged <18 years with or without ARI (ARI; non-ARI, respectively), during March 1, 2020–February 26, 2021, determined using presence or absence of ICD-10-CM codes for COVID-19 and ARI. The non–COVID-19 group was matched 5:1 to the COVID-19 group by age, sex, and month of encounter. The ARI group was matched 5:1 to the COVID-19 group by age and sex, and a random encounter date was selected. The non-ARI group was matched 2:1 to the ARI group by age and sex, and a random encounter date was selected. In HealthVerity, among patients aged <18 years, those with COVID-19 (COVID), determined by ICD-10-CM code or by a positive SARS-CoV-2 test result during March 1, 2020–June 28, 2021, were matched 1:1 to those with a negative SARS-CoV-2 test result (non–COVID-19) during the same period.

^†^ New diabetes diagnosis could occur >30 days after the index encounter and included ICD-10-CM codes E08 (diabetes due to underlying condition), E09 (drug or chemical induced diabetes), E10 (type 1 diabetes), E11 (type 2 diabetes), and E13 (other specified diabetes).

^§^ The denominator was patients aged <18 years who received a new diabetes diagnosis. Diabetes ICD-10-CM codes at the new diabetes encounter were grouped into any type 1 or type 2 code (E10, E11), diabetes due to underlying condition (E08) or other specified diabetes (E13) codes, or drug induced diabetes (E09).

^¶^ The denominator was patients aged <18 years with a new diabetes diagnosis. DKA was defined as E08.1, E09.1, E10.1, E11.1, and E13.1 coded any time before and including the index encounter.

** Dashes indicate no prepandemic data available for ARI and non-ARI in the HealthVerity database.

In the IQVIA database, diabetes incidence was 316 per 100,000 person-years in the COVID-19 group, 118 per 100,000 person-years in the pandemic period non–COVID-19 group, 126 per 100,000 person-years in the prepandemic ARI group, and 125 per 100,000 person-years in the prepandemic non-ARI group (Table 2). Diabetes risk was 166% higher in the COVID-19 group than in the non-COVID-19 group (HR = 2.66, 95% CI = 1.98–3.56) and 116% higher than in the prepandemic ARI group (HR = 2.16, 95% CI = 1.64–2.86) (Figure). Diabetes incidence did not significantly differ between the prepandemic ARI and non-ARI groups (HR = 0.99, 95% CI = 0.84–1.15). In the HealthVerity database, diabetes incidence was 31% higher among patients aged <18 years with COVID-19 (399 per 100,000 person-years) than among those without COVID-19 (304 per 100,000 person-years; HR = 1.31, 95% CI = 1.20–1.44).

**TABLE 2:** Incidence of new diabetes diagnoses by age group and sex — IQVIA PharMetrics Plus and HealthVerity claims databases, United States, March 1, 2020–June 28, 2021*

Database/Characteristic	COVID-19			Non–COVID-19			ARI			Non-ARI		
	No. of DM cases	Person-years	Diabetes incidence^†^ (95% CI)	No. of DM cases	Person-years	Diabetes incidence^†^ (95% CI)	No. of DM cases	Person-Years	Diabetes incidence^†^ (95% CI)	No. of DM cases	Person-years	Diabetes incidence^†^ (95% CI)
**IQVIA**												
**Overall**	68	21,563	316 (241–391)	132	111,418	118 (98–139)	227	180,436	126 (109–142)	510	407,741	125 (114–136)
**Age group, yrs**												
0–11	20	7,662	261 (146–375)	30	39,512	76 (49–103)	56	65,810	85 (63–107)	148	147,255	101 (84–117)
12–17	48	13,886	346 (248–443)	102	71,906	142 (114–169)	171	114,626	149 (127–172)	362	260,486	139 (125–153)
**Sex**												
Female	34	10,849	313 (208–419)	69	56,112	123 (94–152)	125	90,835	138 (113–162)	252	203,209	124 (109–139)
Male	34	10,699	318 (211–425)	63	55,306	114 (86–142)	102	89,601	114 (92–136)	258	204,532	126 (111–142)
**HealthVerity**												
**Overall**	1120	280,767	399 (376–423)	853	281,072	304 (284–324)	—^§^	—	—	—	—	—
**Age group, yrs**												
0–11	240	113,575	211 (186–239)	214	113,642	188 (164–214)	—	—	—	—	—	—
12–17	880	167,192	526 (492 –562)	639	167,430	381 (353–412)	—	—	—	—	—	—
**Sex**												
Female	602	140,844	427 (394–462)	478	141,018	339 (310–370)	—	—	—	—	—	—
Male	518	139,914	370 (339–403)	375	140,045	268 (242–296)	—	—	—	—	—	—

**Abbreviations:** ARI = acute respiratory infection; DM = diabetes mellitus, ICD-10-CM = *International Classification of Diseases, Tenth Revision, Clinical Modification*.

* Groups in IQVIA included patients aged <18 years with or without COVID-19 (COVID-19; non–COVID-19, respectively) and patients aged <18 years with or without ARI (ARI; non-ARI, respectively), during March 1, 2020–February 26, 2021, determined using presence or absence of ICD-10-CM codes for COVID-19 and ARI. The non–COVID-19 group was matched 5:1 to the COVID-19 group by age, sex, and month of encounter. The ARI group was matched 5:1 to the COVID-19 group by age and sex, and a random encounter date was selected. The non-ARI group was matched 2:1 to the ARI group by age and sex, and a random encounter date was selected. In HealthVerity, among patients aged <18 years, those with COVID-19 (COVID), determined by ICD-10-CM code or by a positive SARS-CoV-2 test result during March 1, 2020–June 28, 2021, were matched 1:1 to those with a negative SARS-CoV-2 test result (non–COVID-19) during the same period by age, sex, and month of encounter.

^†^ Cases per 100,000 person-years.

^§^ Dashes indicate no prepandemic data available for ARI and non-ARI in the HealthVerity database.

**FIGURE:**
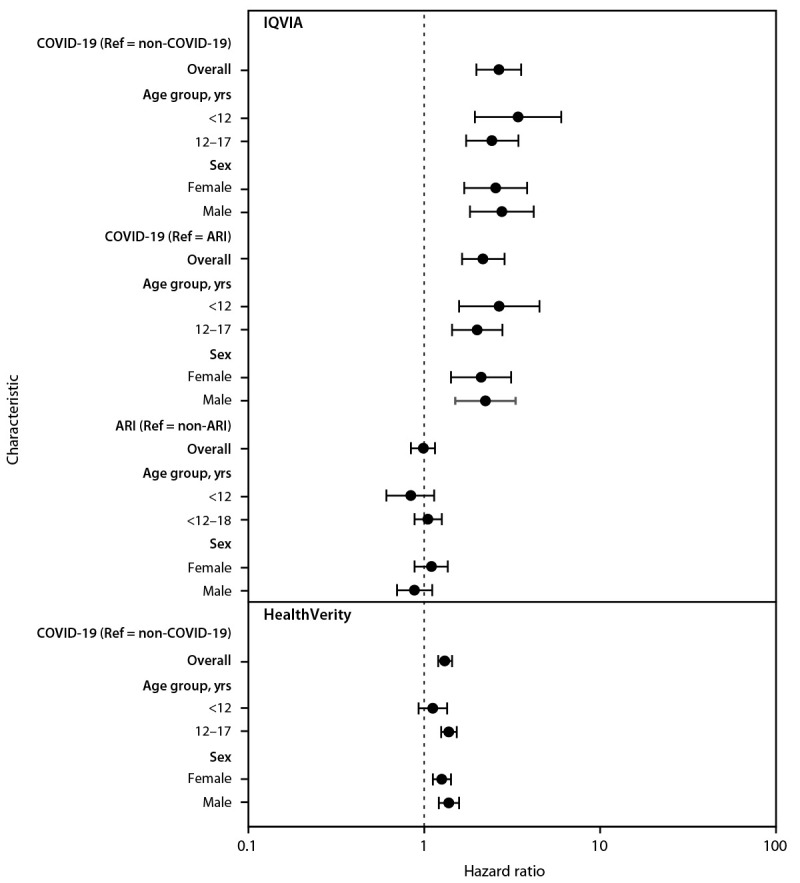
Hazard ratio for the association between COVID-19 or acute respiratory infection and new diabetes diagnosis among patients aged <18 years, by age group and sex — IQVIA PharMetrics Plus and HealthVerity claims databases,* United States, March 1, 2020–June 28, 2021^†^^,^^§^^,^^¶^ **Abbreviations:** ARI = acute respiratory infection; HR = hazard ratio, ICD-10-CM = *International Classification of Diseases, Tenth Revision, Clinical Modification*; Ref = referent. * https://www.iqvia.com/; https://healthverity.com/ ^†^ 95% CIs indicated by error bars. ^§^ Groups in IQVIA included patients aged <18 years with or without COVID-19 (COVID-19; non–COVID-19, respectively) and patients aged <18 years with or without ARI (ARI; non-ARI, respectively), during March 1, 2020–February 26, 2021, determined using presence or absence of ICD-10-CM codes for COVID-19 and ARI. The non–COVID-19 group was matched 5:1 to the COVID-19 group by age, sex, and month of encounter. The ARI group was matched 5:1 to the COVID-19 group by age and sex, and a random encounter date was selected. The non-ARI group was matched 2:1 to the ARI group by age and sex, and a random encounter date was selected. In HealthVerity, among patients aged <18 years, those with COVID-19 (COVID), determined by ICD-10-CM code or by a positive SARS-CoV-2 test result during March 1, 2020–June 28, 2021, were matched 1:1 to those with a negative SARS-CoV-2 test result (non–COVID-19) during the same period by age, sex, and month of encounter. ^¶^ Hazard ratios are plotted on a logarithmic scale.

In the IQVIA database, risk for diabetes was similar across age groups and by sex. In the HealthVerity database, there was no association with diabetes in children aged <12 years, although a significantly increased risk was observed among all other age and sex groups. However, no age group or sex interaction terms were statistically significant.

## Discussion

New diabetes diagnoses were 166% (IQVIA) and 31% (HealthVerity) more likely to occur among patients with COVID-19 than among those without COVID-19 during the pandemic and 116% more likely to occur among those with COVID-19 than among those with ARI during the prepandemic period. Non–SARS-CoV-2 respiratory infection was not associated with diabetes. These findings are consistent with previous research demonstrating an association between SARS-CoV-2 infection and diabetes in adults (*4*–*7*). The inclusion of only patients aged <18 years with a health care encounter possibly related to COVID-19 in the non–COVID-19 HealthVerity group could account for the lower magnitude of increased diabetes risk in this group compared with risk in the IQVIA group. In addition, patients without COVID-19 in HealthVerity had higher hospitalization rates than did those in IQVIA, suggesting more severe disease at the index encounter in the HealthVerity comparison group.

The observed association between diabetes and COVID-19 might be attributed to the effects of SARS-CoV-2 infection on organ systems involved in diabetes risk. COVID-19 might lead to diabetes through direct attack of pancreatic cells expressing angiotensin converting enzyme 2 receptors, through stress hyperglycemia resulting from the cytokine storm and alterations in glucose metabolism caused by infection, or through precipitation of prediabetes to diabetes (*8*). A percentage of these new diabetes cases likely occurred in persons with prediabetes, which occurs in one in five adolescents in the United States.^††††^ Steroid treatment during hospitalization might lead to transient hyperglycemia; however, only 1.5%–2.2% of diabetes codes were for drug- or chemical-induced diabetes, with the majority of codes being for type 1 or type 2 diabetes. Alternatively, COVID-19 might have indirectly increased diabetes risk through pandemic-associated increases in body mass index,^§§§§^ a risk factor for both serious COVID-19 illness and diabetes. Future studies addressing the role of comorbidities and increases in body mass index in post–COVID-19 diabetes are warranted. Although this study can provide information on the risk for diabetes following SARS-CoV-2 infection, additional data are needed to understand underlying pathogenic mechanisms, either those caused by SARS-CoV-2 infection itself or resulting from treatments, and whether a COVID-19–associated diabetes diagnosis is transient or leads to a chronic condition.

Evidence of increased pediatric type 1 diabetes has been reported during the COVID-19 pandemic (*1*,*2*). Among persons aged <18 years with COVID-19 and new diabetes diagnoses in this study, nearly one half had DKA at or around the time of diagnosis. This number was higher than that in comparison groups, and higher than previous reports of DKA among incident type 1 diabetes cases before the pandemic (28%) (*9*). Increased frequency of DKA at time of diagnosis of type 1 diabetes during the pandemic has previously been reported and was thought to be due to delayed care-seeking for diabetes (*3*). However, the observed association of increased risk for diabetes diagnosis following SARS-CoV-2 infection would not be explained solely by delayed care. COVID-19 has disproportionately affected racial/ethnic minority groups, and those aged <18 years in these groups are also at increased risk for type 2 diabetes (*10*). An association between COVID-19 and new pediatric diabetes diagnoses might disproportionately affect racial/ethnic minority groups. Race/ethnicity data were unavailable in the present data sets; however, future studies should address racial and ethnic disparities in COVID-19 and diabetes, and whether persons aged <18 years who are at risk for COVID-19 are also those at risk for delaying medical care.

Health care providers should screen for diabetes symptoms in persons aged < 18 years with a history of SARS-CoV-2 infection. These symptoms can include frequent urination, increased thirst, increased hunger, weight loss, tiredness or fatigue, stomach pain, and nausea or vomiting.^¶¶¶¶^

The findings in this report are subject to at least four limitations. First, the definition of diabetes might have low specificity because it used a single ICD-10-CM code, did not include laboratory data at the time of diagnosis, and could not reliably distinguish between type 1 and type 2 diabetes. Second, patients infected with SARS-CoV-2 without a COVID-19 diagnosis or documented positive test result might be misclassified as not having COVID-19. Third, the present analyses lacked information on covariates that could have affected the association between COVID-19 and incident diabetes, including prediabetes, race/ethnicity, and obesity status. Finally, estimated associations are only representative of persons aged <18 years seeking care included in these commercial claims databases and not of pediatric populations with SARS-CoV-2 infection without commercial health insurance or who do not seek health care.

These data suggest an increased risk for diabetes among persons aged <18 years with COVID-19, which is supported by independent studies in adults (*4*–*7*). These findings underscore the importance of COVID-19 prevention among all age groups, including vaccination for all eligible children and adolescents, and chronic disease prevention and treatment. Public health messages highlighting the risks associated with COVID-19 among the pediatric population are especially important to inform clinicians and parents about possible sequelae of COVID-19. SARS-CoV-2 infection might lead to type 1 or type 2 diabetes through complex and differing mechanisms. Partner agencies and clinicians in the field should be aware of long-term consequences and monitor persons aged <18 years in the months following a SARS-CoV-2 infection for new diabetes onset. Long-term follow-up studies of COVID-19 are warranted to further define the potential association between COVID-19 and increased diabetes risk among those in this age group.

**Summary**

**What is already known about this topic?**

SARS-CoV-2 infection is associated with worsening of diabetes symptoms, and persons with diabetes are at increased risk for severe COVID-19. SARS-CoV-2 infection might also induce newly diagnosed diabetes.

**What is added by this report?**

Persons aged <18 years with COVID-19 were more likely to receive a new diabetes diagnosis >30 days after infection than were those without COVID-19 and those with prepandemic acute respiratory infections. Non–SARS-CoV-2 respiratory infection was not associated with an increased risk for diabetes.

**What are the implications for public health practice?**

The increased diabetes risk among persons aged <18 years following COVID-19 highlights the importance of COVID-19 prevention strategies in this age group, including vaccination for all eligible persons and chronic disease prevention and treatment.

## Footnotes

*
https://www.cdc.gov/coronavirus/2019-ncov/hcp/clinical-care/underlyingconditions.html

† CDC defines post–COVID-19 conditions as new, returning, or ongoing health problems occurring ≥4 weeks after being infected with SARS-CoV-2. https://www.cdc.gov/coronavirus/2019-ncov/long-term-effects/index.html

§ As of January 7, 2021, children aged ≥5 years are eligible for COVID-19 vaccination. https://www.cdc.gov/vaccines/covid-19/clinical-considerations/covid-19-vaccines-us.html

¶ IQVIA PharMetrics Plus (https://www.iqvia.com) is a database of adjudicated health care claims from closed U.S. health plans, including claims from primarily commercial health plans (preferred provider and health maintenance) used to provide a complete view of patient care across all care settings. During January 2013–March 2021, PharMetrics Plus had approximately 163 million enrollees. IQVIA data (2021 Quarter 3 2021 data release) were extracted using the E360 Software-as-a-Service Platform. https://www.iqvia.com/solutions/real-world-evidence/platforms/e360-real-world-data-platform.

** HealthVerity (https://healthverity.com/) provides access to patient-level linked data from 70 different commercial health data sources using privacy-preserving record linkage to generate a comprehensive and longitudinal patient history. During 2014–2021, there were medical claims of approximately 150 million patients. This study used CDC-licensed HealthVerity (November 2021 data release) closed payor claims data linked to SARS-CoV-2 laboratory testing and hospital chargemaster data for patients with any health care encounter possibly related to COVID-19.

†† In IQVIA, the study period was 2 years and 2 months (January 29, 2019–March 31, 2021) for the pandemic period groups or January 29, 2016–March 31, 2018 for the prepandemic period groups. In HealthVerity, the study period was December 1, 2018–July 31, 2021.

§§ The index date for the COVID-19 group was the first outpatient claim or hospital discharge date with a COVID-19 diagnosis (IQVIA, HealthVerity) or a positive SARS-CoV-2 test result (HealthVerity). The index date for the non–COVID-19 group was the date of a randomly selected claim during the month in which the patient was matched to a COVID-19 group patient (IQVIA and HealthVerity). Because of a lack of ARI cases in winter months comparable to COVID-19, the index dates for the ARI and non-ARI groups were defined based on a randomly chosen ARI or non-ARI claim during the prepandemic study period (IQVIA).

¶¶ ICD-10-CM B97.29 code (other coronavirus as the cause of diseases classified elsewhere) between March–April 2020 and U07.1 code (COVID-19, virus identified [laboratory-confirmed]) beginning April 2020. https://www.cdc.gov/nchs/data/icd/Announcement-New-ICD-code-for-coronavirus-3-18-2020.pdf

*** The maximum possible matching ratio was used in each comparison. In IQVIA, the non–COVID-19 and ARI groups were both matched 5:1 to the COVID-19 group and the non-ARI group was matched 2:1 to the ARI group. In HealthVerity, the non–COVID-19 group was matched 1:1 to the COVID-19 group.

††† ICD-10-CM diagnoses related to COVID-19 and multisystem inflammatory syndrome in children (MIS-C) that were used to exclude possible COVID-19 in non–COVID-19 groups include B97.29, U07.1, B34.2, B97.2, B97.21, J12.82, U07.2, A41.89, J12.81, J12.89, M35.8, B94.8, M30.3, and M35.81.

§§§ Among those with ARI, the most common ARI codes were acute pharyngitis (J02, 38.3%), acute upper respiratory infection of multiple and unspecified sites (J06, 22.1%), acute sinusitis (J01, 11.8%), influenza due to unidentified influenza virus (J10, 4.8%), influenza due to other identified influenza virus (J11, 4.5%), acute bronchitis (J20, 4.3%), acute tonsilitis (J03, 3.1%), and acute nasopharyngitis (common cold) (J00, 2.7%).

¶¶¶ Frequencies of incident diabetes codes within the following categories were calculated: type 1 diabetes or type 2 diabetes (E10-E11), diabetes due to underlying condition or other diabetes (E08, E13), and drug or chemical induced diabetes (E09). DKA was defined as E08.1, E09.1, E10.1, E11.1, and E13.1 coded before or including the incident diabetes encounter.

**** 45 C.F.R. part 46, 21 C.F.R. part 56; 42 U.S.C. Sect. 241(d); 5 U.S.C. Sect. 552a; 44 U.S.C. Sect. 3501 et seq.

††††
https://www.cdc.gov/media/releases/2019/p1202-diabetes.html

§§§§
https://www.cdc.gov/mmwr/volumes/70/wr/mm7037a3.htm?s_cid=mm7037a3_w

¶¶¶¶
https://www.cdc.gov/diabetes/basics/symptoms.html
